# Identification of Crucial Genes Associated With MYCN‐Driven Neuroblastoma Based on Single‐Cell Analysis and Machine Learning

**DOI:** 10.1002/cam4.71008

**Published:** 2025-07-02

**Authors:** Jiasi Zhang, Yichen Lei, Yaqin Wang, Wen Yu, Xiaoyan Zhao, Yongbing Zhu, Dedong Zhang, Siying Liu, Aiguo Liu

**Affiliations:** ^1^ Department of Pediatric Tongji Hospital, Tongji Medical College, Huazhong University of Science and Technology Wuhan China; ^2^ Key Laboratory of Molecular Biological Targeted Therapies of the Ministry of Education, Huazhong University of Science and Technology China

**Keywords:** MYCN amplification, neuroblastoma, nomogram model, random survival forests, single‐cell transcriptome

## Abstract

**Background:**

Neuroblastoma (NB) with MYCN amplification is strongly correlated with high‐risk stratification and poor prognosis. However, the underlying mechanisms remain incompletely understood. Elucidating these pathways is critical for advancing personalized treatments for MYCN‐driven NB.

**Methods:**

We performed single‐cell transcriptomic analysis comparing NB samples with and without MYCN. Key genes were then identified using machine learning based random survival forest (RSF) and nomogram analyses. The influence of key genes on immune infiltration and molecular mechanisms driving NB progression were further investigated. Finally, we visualized the expression levels and global function of these genes in single‐cell datasets and validated their expression in patient samples through RT‐qPCR.

**Results:**

Single‐cell transcriptome analysis of GSE218450 identified marker genes specific to NB cells. RSF and nomogram analyses revealed that overexpression of CKB, PCSK1N, OTUB1, and VGF is associated with poor prognosis, whereas upregulation of NTRK3 indicates a favorable prognosis. These genes are significantly associated with immune cell infiltration and play an important role in modulating the immune microenvironment. Pathway analysis further showed that these genes influence critical signaling pathways, including the Wnt pathway, and interact with tumor‐related genes. Additionally, we confirmed that CKB and PCSK1N are positively correlated with MYCN in NB cell lines and are significantly overexpressed in MYCN‐amplified NB patients.

**Conclusions:**

Our results provide molecular insights into the transcriptional changes associated with MYCN amplification in NB. In particular, the identification of CKB and PCSK1N suggests their potential role in driving tumor progression, making them promising targets for novel treatments in MYCN‐driven NB.

## Graphical abstract

By analyzing single‐cell transcriptomic data from 5 neuroblastoma patients with MYCN amplification and 7 patients without MYCN amplification (GSE21840), and applying machine learning approaches, we identified five candidate pathogenic genes potentially associated with MYCN amplification. These genes are significantly correlated with immune cell infiltration and appear to play pivotal roles in modulating the tumor immune microenvironment. Pathway enrichment analysis further revealed their involvement in key signaling pathways, including the Wnt pathway. Notably, CKB and PCSK1N emerged as potential drivers of tumor progression, highlighting their promise as therapeutic targets in MYCN‐amplified neuroblastoma.

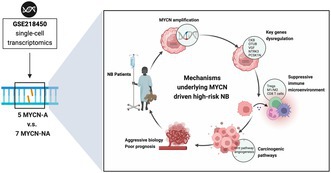

**Abbreviations**
AUC: area under the curve
CCLE: Cancer Cell Line Encyclopedia
CKB: creatine kinase B
DCA: decision curve analysis
E2F4: E2F transcription factor 4
GEO: Gene Expression Omnibus
GO: Gene Ontology
GSEA: Gene Set Enrichment Analysis
GSVA: Gene Set Variation Analysis
KEGG: Kyoto Encyclopedia of Genes and Genomes
MAD: Median Absolute Deviation
MHC: major histocompatibility complex
NB: neuroblastoma
NCCN: National Comprehensive Cancer Network
NES: normalized enrichment score
NK: natural killer
NK: natural killer
NTRK3: neurotrophin receptor tyrosine kinase 3
OTUB1: OTU domain‐containing ubiquitin aldehyde‐binding protein 1
PAX5: Paired box 5
PCA: principal component analysis
PCSK1N: proprotein convertase subtilisin/kexin type 1 inhibitor
RSF: random survival forest
RT‐qPCR: real‐time quantitative reverse transcription PCR
scRNA‐Seq: single‐cell RNA sequencing
SOX17: SRY‐Box Transcription Factor 17
TCGA: Cancer Genome Atlas Program
Tregs: regulatory T cells
UMAP: Uniform Manifold Approximation and Projection
UMI: Unique Molecular Identifier
VGF: nerve growth factor inducible

## 1 Introduction

Neuroblastoma (NB) is a type of cancer that arises from immature nerve cells, typically originating from adrenal glands but can also develop in the neck, chest, abdomen, or spine [1]. It is the most common extracranial solid tumor in children and accounts for about 6% of all childhood cancers, with the majority of cases diagnosed before the age of 5 [2]. NB exhibits a broad spectrum of clinical behaviors, ranging from spontaneous regression in low‐risk cases to aggressive, treatment‐resistant forms in high‐risk cases [3]. Prognosis and treatment strategies are largely determined by risk stratification, which is influenced by factors such as age at diagnosis, tumor stage, histopathology characteristics, and genetic markers. Among these genetic markers, MYCN amplification is particularly significant, which is observed in approximately 20%–25% of all NB cases [4]. Accumulating evidences have shown that MYCN‐amplified NB are less responsive to treatments such as chemotherapy and radiation therapy [5]. Therefore, children with MYCN amplification still face poor outcomes [6].

MYCN amplification has a wide‐ranging and profound effect on tumor biology of NB, including oncogene activation, metabolic reprogramming, epigenetic modulation, and interaction with signal pathways like PI3K/AKT/mTOR and MAPK pathways [7, 8]. Particularly, MYCN upregulates DNA repair genes, reduce apoptosis, and increase the expression of drug efflux pumps to promote treatment resistance [9, 10], even modulates immune microenvironment by downregulating the expression of immune‐stimulatory molecules and upregulating immune checkpoint proteins, helping NB cells evade immune surveillance [11]. Indeed, emerging evidences have shown that MYCN drives an immunosuppressive environment, including reduced natural killer (NK) cell cytotoxicity, dysfunctional T‐cell profile, and immunosuppressive myeloid population, these impair anti‐tumor immunity and impact NB patient survival [11, 12]. Whereas, the signaling pathways of MYCN are complex and not fully understood, involving numerous genes in cell proliferation, differentiation and interactions with immune cells, making it challenging to pinpoint specific therapeutic targets.

Single‐cell RNA sequencing (scRNA‐Seq) has emerged as a powerful tool to dissect the complexity of cancer at an unprecedented resolution, allowing for a more detailed and precise understanding of tumor biology by providing data at the individual cell level [13]. The application of scRNA‐Seq in studying MYCN amplification is particularly promising in NB. First, NB is characterized by significant heterogeneity, with subpopulation cells showing different levels of MYCN expression and varying genetic and epigenetic profiles [14]. ScRNA‐Seq helps map this heterogeneity, providing insights into how MYCN amplification drives NB evolution, including identify distinct subpopulations that survive treatment and elucidate the specific molecular changes that confer drug resistance. Second, scRNA‐Seq allows for the characterization of not only cancer cells but also the surrounding stromal, immune, and endothelial cells [15]. This helps to understand the interactions between MYCN‐amplified NB cells and the tumor microenvironment, especially for immune cells. It can reveal how MYCN influences immune evasion and shapes the immune landscape [12].

Based on the results of single‐cell dataset analysis, survival analysis focusing on NB might help us screen key genes associated with MYCN. Random Survival Forest (RSF) is suitable for survival analysis because of its ability to deal with missing data. RSF can capture complex non‐linear relationships and analyze intricate interactions within gene expression data [16]. Additionally, RSF provides variable importance scores, allowing us to identify genes with significant impacts on survival outcomes, which is particularly valuable for screening crucial oncogenes [17]. In contrast, other methods such as Artificial Neural Networks (ANN), Support Vector Machines (SVM), and Deep Learning (DL/DNN) lack these advantages. Besides, nomogram models are graphical representations that predict the probability of a particular outcome based on multiple variables [18]. The principles involve regression techniques, typically using logistic or Cox proportional hazards models, to quantify the relationship between predictors and disease outcome [19]. Applications range from predicting patient survival rates to assessing the likelihood of treatment response, providing a visual tool for clinicians to personalize patient care.

In this study, we employ RSF to identify the crucial genes associated with MYCN amplification in NB single‐cell transcriptomics. Concurrently, bulk transcriptome provides a comprehensive understanding of potential pathways of crucial genes and the influence on immune infiltration and immune checkpoint. The survival analysis was further conducted using univariate and multivariate nomogram prediction models. Then the expression of key genes in the single‐cell dataset was visualized and the correlations between NB pathogenic genes and crucial genes were analyzed. Lastly, the expression levels of crucial genes were validated by experimental results. Overall, our study offers a novel perspective on the diverse and complex role of MYCN in NB, emphasizing poor prognostic genes linked to MYCN and elucidating their potential mechanisms.

## 2 Methods

### 2.1 Data Acquisition

The public NB‐related dataset was obtained from the Gene Expression Omnibus (GEO) database (https://www.ncbi.nlm.nih.gov/geo/), with accession number GSE218450 [20]. Our study comprises a total of 12 samples, including 5 MYCN‐amplified and 7 MYCN non‐amplified patients. Additionally, bulk mRNA data were downloaded from the Cancer Genome Atlas (TCGA) database (https://www.cancer.gov/ccg/). This dataset includes expression profiles from 159 NB patients, including 32 MYCN‐amplified and 127 MYCN non‐amplified samples. Besides, the expression levels of crucial genes in NB cell lines were obtained from the Cancer Cell Line Encyclopedia (CCLE) (https://sites.broadinstitute.org/ccle).

### 2.2 Quality Control of Single‐Cell Dataset

First, we imported the expression profile using the Seurat package, filtering low‐quality cells based on total unique molecular identifier (UMI) counts, the number of expressed genes, and the percentages of mitochondrial and ribosomal gene expression. Cells with high mitochondrial and ribosomal gene expression often exhibit low RNA expression levels, suggesting they may be undergoing cell death. To ensure rigorous quality control, we applied the Median Absolute Deviation (MAD) method, following the guideline that values exceeding 3 MAD from the median are considered outliers and removed. Additionally, DoubletFinder (V2.0.4) was used to filter out doublets in each sample, completing the quality control process. Thes approaches help maintain the integrity and reliability of analyzed dataset.

### 2.3 Clustering and Annotation of Single‐Cell Data

Second, we applied the LogNormalize method for global normalization, adjusting the total expression of each cell to 10,000 by multiplying it by a scaling factor, followed by logarithmic transformation for normalization. Then CellCycleScoring was used to calculate cell cycle scores, and FindVariableFeatures was applied to identify highly variable genes, and ScaleData function to account for gene expression fluctuations caused by mitochondrial and ribosomal gene expression proportions, as well as cell cycle stage. To perform linear dimensionality reduction, we applied RunPCA, selecting 20 principal components for principal component analysis (PCA). Harmony was utilized to correct for batch effects, while RunUMAP facilitated nonlinear dimensionality reduction through Unified Manifold Approximation and Projection (UMAP), with a neighborhood size of 0.2. To identify cell types and their corresponding marker genes, we queried the CellMarker database (http://117.50.127.228/CellMarker/) and PanglaoDB database (https://panglaodb.se/). This was further supplemented by relevant literature and automated annotation using the SingleR software. Finally, we used the FindAllMarkers function to identify marker genes for each cell type.

### 2.4 Functional Enrichment Analysis

To elucidate the signaling pathways associated with differential expression genes, we utilized R package “ClusterProfiler” for functional annotation. This approach allowed us to comprehensively explore the relevance of these genes. Then we assessed the functional pathways using Gene Ontology (GO) (https://www.geneontology.org/) and Kyoto Encyclopedia of Genes and Genomes (KEGG) (https://www.genome.jp/kegg/). Significance of enriched GO terms and KEGG pathways was determined based on both *p*‐values and *q*‐values, with thresholds set at less than 0.05 for statistical significance.

### 2.5 Random Survival Forest Analysis

Feature selection was conducted using the randomForestSRC package, which allowed us to leverage the random survival forest algorithm for ranking the importance of prognosis‐related genes. In this analysis, we set the number of iterations in the Monte Carlo simulation to 1000 (nrep = 1000) to ensure robust results. By evaluating the relative importance of each gene, we identified those with a relative importance score greater than 0.8 as our final marker genes. This rigorous approach not only highlights the most impactful genes associated with prognosis but also enhances the reliability of our findings, making them valuable for further research [21].

### 2.6 Nomogram Models

Nomograms are graphical representations constructed based on regression analysis that integrate gene expression levels and clinical symptoms to illustrate their relationships within a predictive model. These diagrams employ lines with scales to display each variable on a common plane in proportion to their contributions [19]. By developing a multifactor regression model, we assign scores to each influencing factor according to its degree of impact on the outcome variable, which is determined by the magnitude of the regression coefficients. The individual scores are then summed to produce a total score, which can be used to calculate the predicted value. This approach facilitates a more intuitive understanding of how various factors contribute to patient outcomes, enhancing the applicability of analyzed models in clinical decision‐making.

### 2.7 Analysis of Immune Cell Infiltration

The CIBERSORT method is a widely utilized approach for assessing immune cell types within the microenvironment. It is based on support vector regression and performs deconvolution analysis on the expression matrix of immune cell subtypes. The method encompasses 547 biomarkers and differentiates 22 human immune cell phenotypes, including T cells, B cells, plasma cells, and various myeloid cell subsets [22]. In this study, we employed the CIBERSORT algorithm to analyze patient data, inferring the relative proportions of the 22 immune infiltrating cells. Additionally, correlation analyses between gene expression profiles and immune cell contents were conducted using CIBERSORT results.

### 2.8 Gene Set Enrichment Analysis

GSEA is frequently utilized to delve deeper into the intersection of tumor classification and biological relevance, thereby enhancing understanding of biological significance within tumor subtypes. GSEA was employed to analyze differential signaling pathways between high and low expression groups [23]. The background gene set utilized was version 7.0 from the MsigDB database, serving as the annotated gene set for subtype pathways. We conducted differential expression analysis of pathways across subtypes, ranking significantly enriched gene sets (adjusted *p* < 0.05) according to their consistency scores.

### 2.9 Gene Set Variation Analysis

Gene Set Variation Analysis (GSVA) is a non‐parametric, unsupervised method for assessing gene set enrichment within the transcriptome. It translates gene‐level changes into pathway‐level alterations by scoring gene sets of interest, helping to determine the biological functions of the samples [23]. In this study, we downloaded gene sets from the Molecular Signatures database (https://www.gsea‐msigdb.org/gsea/msigdb) and applied the GSVA algorithm to comprehensively score each gene set, evaluating potential biological function changes across different samples.

### 2.10 Transcriptional Regulation Analysis of Key Genes

We used R package “RcisTarget” to predict transcription factors, with all calculations being motif‐based. The normalized enrichment score (NES) of a motif is determined by the total number of motifs in the database. Additionally, we inferred annotation files based on motif similarity and gene sequences. The first step in estimating the overexpression of each motif in the gene set involves calculating the area under the curve (AUC) for each motif‐motif set, based on the recovery curve of motif order within the gene set. The NES for each motif is then computed from the AUC distribution of all motifs in the gene set [24].

### 2.11 Patient Samples

Six NB with MYCN and 6 NB without MYCN patients were recruited from Tongji Hospital, Tongji medical College, Huazhong University of Science and Technology (Wuhan, China) between September 2022 and March 2024. NB patients were diagnosed according to the National Comprehensive Cancer Network (NCCN) criteria in February 2024 and had not received any previous treatment. Fresh NB tumor tissue was obtained from the patient after surgery. In a sterile environment, dissect the tumor into small pieces, and incubate the tissue at 37°C for 30 min with agitation. Filter the digested mixture through a cell strainer (70 μm) to remove large debris. Centrifuge the filtered cell suspension at 1000 rpm for 5 min and resuspend the cell pellet in fresh culture medium (DMEM with 10% FBS and 1% antibiotics) [25].

### 2.12 Total RNA Extraction and Real‐Time Reverse Transcription‐PCR

Total RNA was extracted using TRIzol from TaKaRa (TaKaRa, Otsu, Japan). The expression levels of CKB and PCSK1N were detected by RT‐qPCR in using ChamQ SYBR qPCR Master Mix from Vazyme (Vazyme, Nanjing, China). Each RT‐qPCR experiment was performed in triplicate, and gene expression levels were analyzed using the 2^−ΔΔCT^ method, as described in previous studies [26, 27]. β‐actin was employed as the internal reference. All primers were synthesized by Tsingke Biotechnology (Beijing, China) and their sequences are provided in Table S1.

### 2.13 Statistical Analysis

The R language (version 4.3.0) was used for the bioinformatic analysis. The experimental data stems from at least three independent experiments. The experimental results are presented as means ± standard deviation (SD). Unpaired t‐tests were used to compare differences between independent groups. All statistical tests were two‐tailed, and *p* < 0.05 was considered statistically significant.

## 3 Results

### 3.1 Significant Gene Expression Differences in NB Samples With and Without MYCN Amplification Revealed by scRNA‐Seq Analysis

**FIGURE 1:**
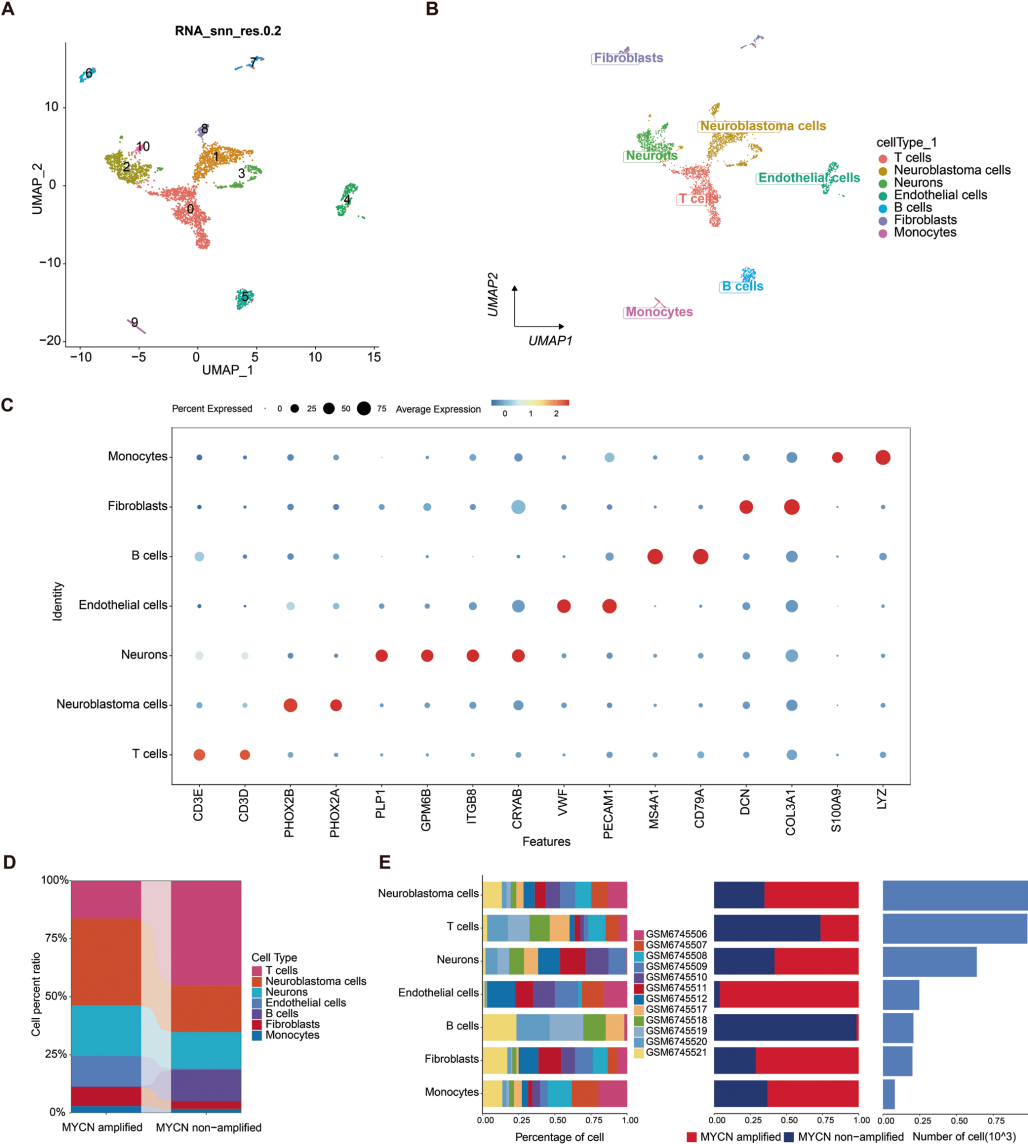
Uniform Manifold Approximation and Projection (UMAP) analysis and cell types annotations between MYCN‐amplified and non‐amplified NB samples. A total of 12 NB samples (5 MYCN‐amplified and 7 MYCN non‐amplified) was obtained from GEO database, with accession number GSE218450. (A) The spatial relationships of 10 distinct cell clusters were visualized using UMAP analysis. (B) Annotation assigned 10 clusters into seven distinct cell types: T cells, neuroblastoma cells, neurons, endothelial cells, B cells, fibroblasts, and monocytes. (C) A bubble chart displaying classic markers of seven cell types. (D) A bar chart comparing seven cell proportions between MYCN‐amplified and non‐amplified groups. (E) A bar chart showing seven cell proportions in 12 NB samples.

To investigate the potential molecular mechanisms of MYCN through scRNA‐Seq, we analyzed a public NB‐related dataset from the GEO database with accession number GSE218450, comprising 5 MYCN‐amplified and 7 MYCN non‐amplified samples. We first conducted quality control, dimensionality reduction, and clustering on the original sequencing data. To ensure data quality across multiple samples, we filtered out cells with fewer than 200 captured genes using the following criteria: (nFeature RNA > 200 and percent.mt ≤ median+3 MAD and nFeature RNA ≤ median+3 MAD and nCount RNA ≤ median+3 MAD). Here, nFeature RNA indicates the number of genes, nCount RNA represents the total UMI count of cells, and percent.mt refers to the percentage of mitochondrial reads. Ultimately, 3337 cells were retained, and violin and scatter plots were generated post‐filtering (Figure S1A,B). We identified 2000 highly variable genes (Figure S1C) and subsequently standardized, normalized, and analyzed the data using PCA, Harmony (Figure S1D–F), and UMAP analysis (Figure 1A).

We further annotated each subtype, identifying all clusters as belonging to seven distinct cell types: T cells, NB cells, neurons, endothelial cells, B cells, fibroblasts, and monocytes (Figure 1B). A bubble chart displaying classical markers for these seven cell types confirmed the reliability of our annotation process (Figure 1C). Additionally, a bar chart comparing cell proportions between MYCN‐amplified and non‐amplified groups revealed significant differences, particularly in NB cells, *T* cells, endothelial cells, B cells, and fibroblasts. Specifically, MYCN‐amplified tumors are characterized by a higher presence of endothelial cells and fibroblasts, with a notable absence of B cell infiltration. In contrast, non‐MYCN‐amplified tumors show B cell infiltration but lack endothelial cells (Figure 1D,E). These findings suggest that MYCN amplification significantly influences the tumor microenvironment and the biological behavior of NB. We utilized the FindAllMarkers function to extract marker genes from the single‐cell data, focusing on genes with an adjusted *p* < 0.05 and a log fold change (logFC) > 0.585 in NB cells (Tables S2 and S3). Then, the differentially expressed genes were obtained by comparing the MYCN amplification with the MYCN non‐amplification group for further analysis (Table S3).

### 3.2 Key Genes Identified by Random Survival Forest and Survival Analysis

**FIGURE 2:**
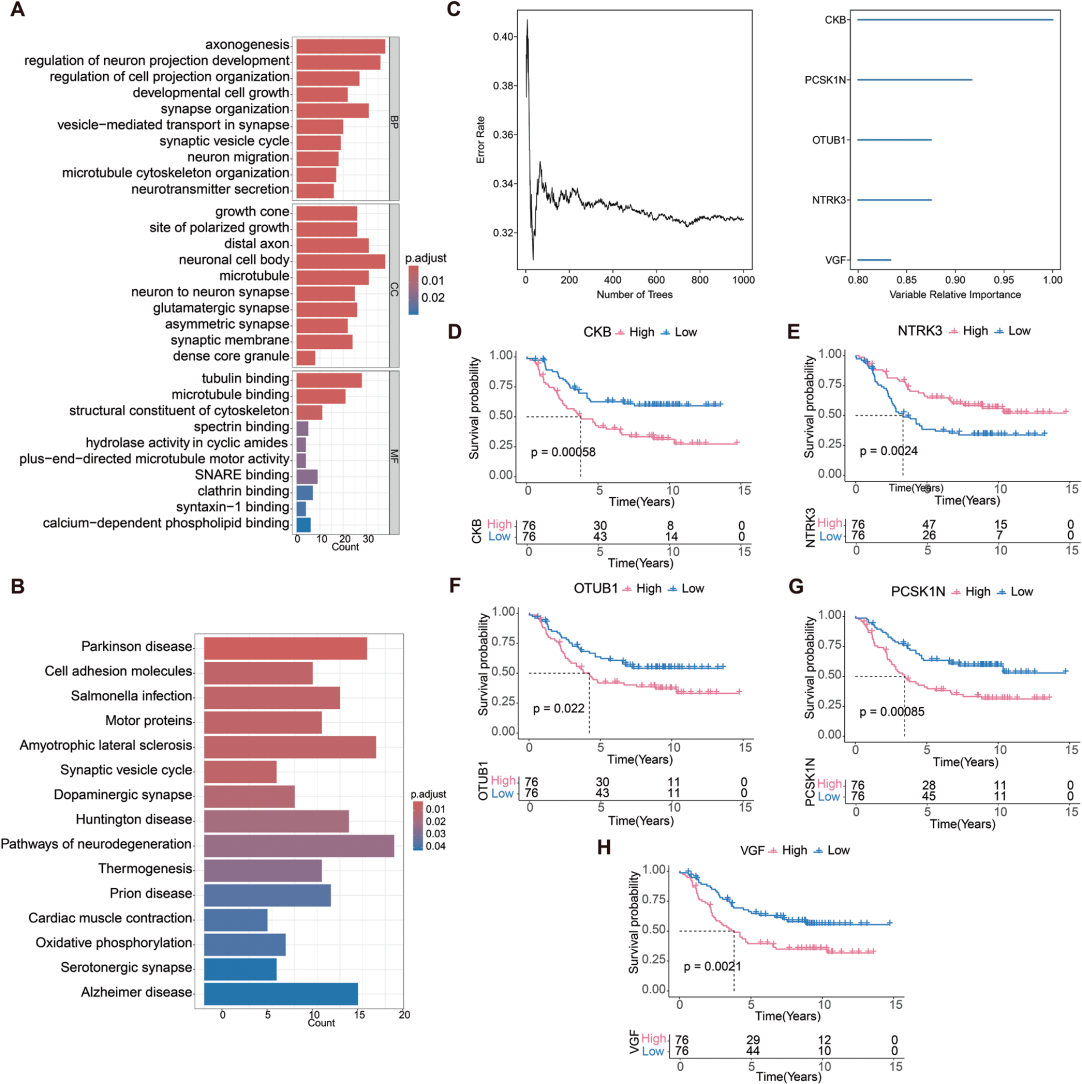
Key genes associated with MYCN amplification were identified by random survival forest (RSF) and survival analyses. 405 marker genes of NB cells were employed for pathway analysis. (A) Gene Ontology (GO) analysis revealed enrichment in pathways such as the regulation of neuron projection development, developmental cell growth, and the synaptic vesicle cycle. (B) Kyoto Encyclopedia of Genes and Genomes (KEGG) analysis indicated enrichment in pathways including cell adhesion molecules, oxidative phosphorylation, and the cell cycle pathway. (C) Marker genes were analyzed by the RSF method to identify key genes associated with poor prognosis in NB. Genes with a relative importance score greater than 0.8 were designated as final markers, and the order of importance was illustrated in right figure. (D‐H) Survival analyses were performed on the five high‐importance genes, including creatine kinase B (CKB) (D), neurotrophin receptor tyrosine kinase 3 (NTRK3) (E), OTU domain‐containing ubiquitin aldehyde‐binding protein 1 (OTUB1) (F), proprotein convertase subtilisin/kexin type 1 inhibitor (PCSK1N) (G), and nerve growth factor inducible (VGF) (H).

We conducted pathway analysis using marker genes, which might reveal insights into gene function. The GO results indicated that these genes were predominantly enriched in pathways associated with the regulation of axonogenesis, neuron projection development, developmental cell growth, and the synaptic vesicle cycle (Figure 2A) (Table S4). The finding suggests a critical role for these genes in neurodevelopmental processes and synaptic function. Furthermore, the KEGG analysis demonstrated that these genes were notably enriched in pathways related to cell adhesion molecules, oxidative phosphorylation, and the cell cycle (Figure 2B) (Table S5). The results underscore the importance of these pathways in cellular communication, energy metabolism, and the regulation of cell division, highlighting potential avenues for further exploration in understanding the underlying biological mechanisms of MYCN.

Given that patients with MYCN amplification are typically classified as high risk [6], we aimed to identify MYCN‐associated key genes that contribute to high‐risk disease progression. Therefore, we analyzed marker genes using the RSF method, a machine learning technique based on decision trees. RSF is widely employed in survival analysis to predict patient survival probabilities, evaluate prognostic risk factors, and select important prognostic‐related genes [28]. We utilized the randomForestSRC package for feature selection and employed the RSF algorithm to rank the importance of prognosis‐related genes (with nrep set to 1000 for Monte Carlo simulations). Genes with a relative importance score greater than 0.8 were designated as final markers, and the order of importance was illustrated (Figure 2C).

Subsequently, we performed survival analysis on these five high‐importance genes, revealing significant associations with survival outcomes (*p* < 0.05). Notably, creatine kinase B (CKB), proprotein convertase subtilisin/kexin type 1 inhibitor (PCSK1N), OTU domain‐containing ubiquitin aldehyde‐binding protein 1 (OTUB1), neurotrophin receptor tyrosine kinase 3 (NTRK3), and nerve growth factor inducible (VGF) were identified as key genes to show a better or worse connections with the outcomes of NB patients (Figure 2D–H). Specifically, the overexpression of CKB, PCSK1N, OTUB1, and VGF is closely linked to poor prognosis, whereas increased NTRK3 expression predicts a favorable prognosis in NB. These five genes were subsequently used as key targets for further research.

### 3.3 Univariate, Multivariate Cox Regression and Nomogram Model Analyses

**FIGURE 3:**
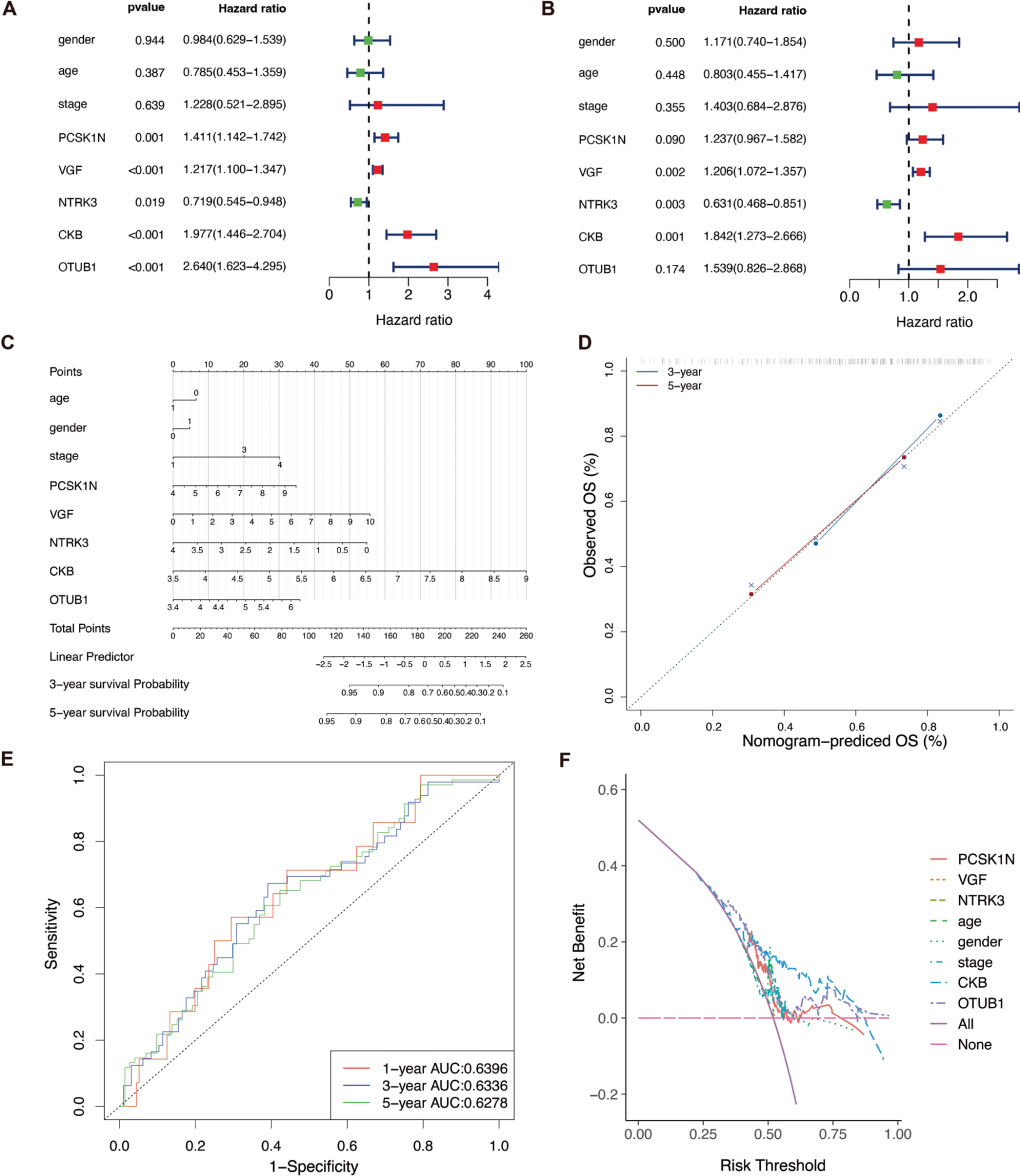
Single‐factor, multifactorial, and nomogram prediction model analyses. (A) The single‐factor Cox regression analyses on key genes including PCSK1N, VGF, NTRK3, CKB, and OTUB1. Hazard ratio > 1 indicates a risk factors, while hazard ratio < 1 indicates a protective factor. (B) The multifactorial Cox regression analyses on key genes including PCSK1N, VGF, NTRK3, CKB, and OTUB1. Hazard ratio > 1 indicates a risk factors, while hazard ratio < 1 indicates a protective factor. (C) Logistic regression analyses were performed by two groups, high and low expression, based on the median values of key genes. (D) Predictive analyses were performed for both 3‐year and 5‐year outcomes in NB. (E) Receiver operating characteristic (ROC) curves were plotted with the corresponding area under curve (AUC) values. (F) Decision curve analysis (DCA) results were conducted on key genes including PCSK1N, VGF, NTRK3, CKB, and OTUB1.

In this study, we established both univariate and multivariate Cox regression models using clinical data and key gene expression levels, accompanied by forest plots to visualize the results. Univariate Cox regression analysis is a survival analysis method used to evaluate the impact of a single factor on patient survival or recurrence risk. It is based on the Cox proportional hazards model, which quantifies the effect of a factor by estimating the hazard ratio. The hazard ratio represents the relative risk of an event occurring between two groups of patients, with values greater than 1 indicating a higher risk and values less than 1 indicating a lower risk [19]. The univariate Cox regression analysis revealed that OTUB1, CKB, VGF, and PCSK1N were identified as risk factors (hazard ratio > 1), while NTRK3 was categorized as a protective factor (hazard ratio < 1) (Figure 3A). Additionally, multivariate Cox regression analysis extends the Cox proportional hazards model to assess the impact of multiple factors on patient survival or recurrence risk. This approach allows for the simultaneous evaluation of multiple predictor variables while controlling for the influence of other factors [29]. In the multifactorial Cox regression analysis, OTUB1, CKB, VGF, and PCSK1N remained as risk factors (hazard ratio > 1), and NTRK3 continued to function as a protective factor (hazard ratio < 1) (Figure 3B), which supports the reliability of our preliminary research and analysis results.

To further elucidate the impact of key gene expression, we divided the samples into two groups, high and low expression, based on the median values of key genes. The result of regression analysis was subsequently presented in the form of column line graphs. Notably, logistic regression analysis indicated that the expression levels of key genes significantly contributed to the scoring process of the nomogram prediction model (Figure 3C). Then we performed predictive analyses for both 3‐year and 5‐year outcomes in NB (Figure 3D). We plotted receiver operating characteristic (ROC) curves and acquired the corresponding AUC values (Figure 3E). AUC is a numerical representation of the area under the ROC curve. It serves as a quantitative measure of a classification model's overall performance. AUC values range from 0 to 1, with higher values indicating better model performance. The ROC curve measures the diagnostic accuracy of a prediction model but does not consider the clinical utility of the model. In contrast, decision curve analysis (DCA) is valuable for assessing the application of models in actual clinical decision‐making [21]. So DCA curves were analyzed as well, showing that our previous prediction model is of practical value (Figure 3F). These comprehensive analyses not only reinforced the reliability of the five key prognostic genes but also demonstrated their potential utility in clinical prediction models. Overall, our findings emphasize the importance of integrating gene expression data with clinical factors to enhance predictive accuracy in NB patient outcomes.

### 3.4 The Impact of Key Genes on Immune Infiltration and Associations

**FIGURE 4:**
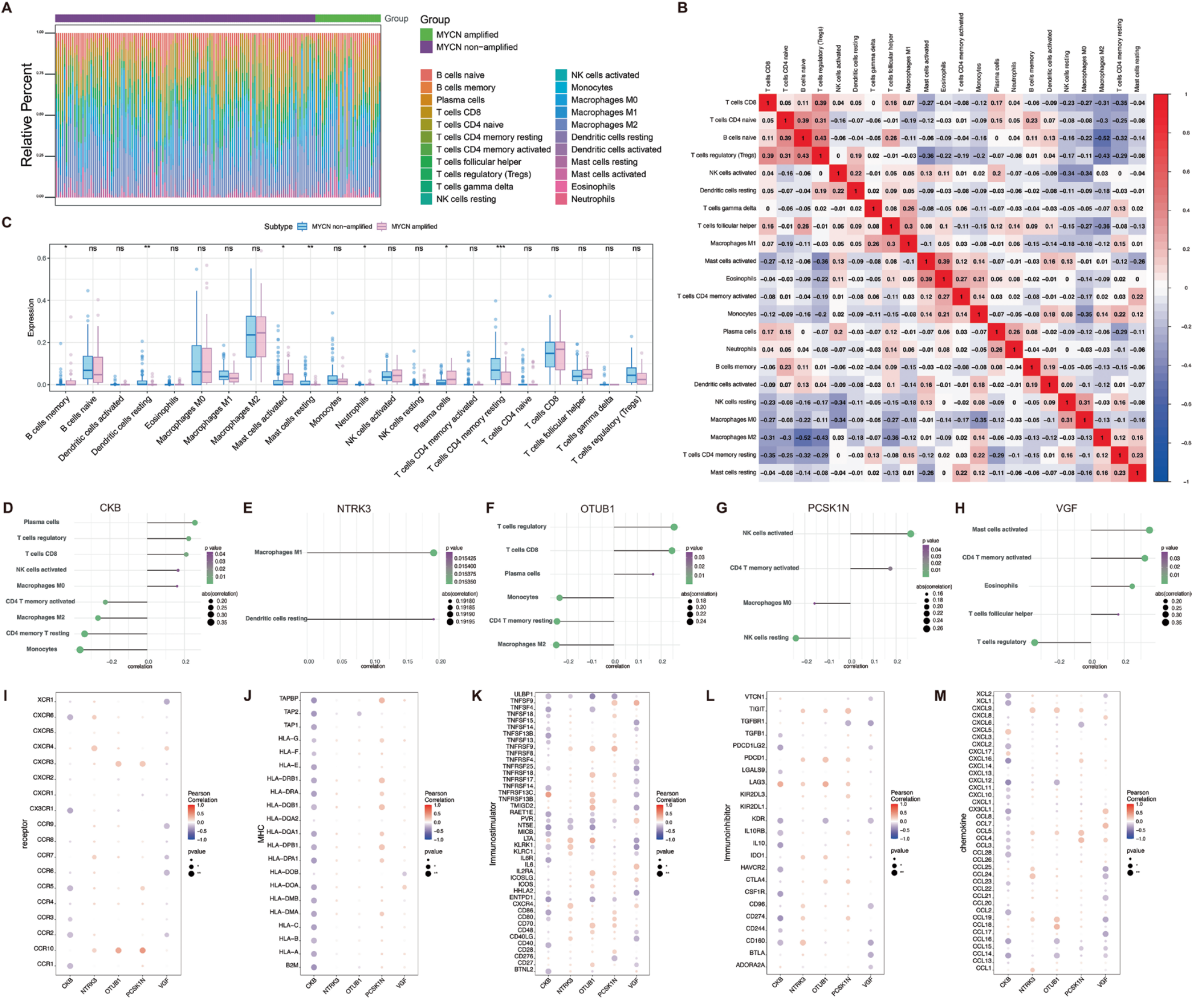
The impact of key genes on immune infiltration and associations. (A‐B) The proportions of immune cell content in NB patients with and without MYCN amplification (A), as well as the correlations among immune cells (B). (C) The differences in the levels of various immune cells between the MYCN‐amplificated and non‐amplificated groups. (D–H) The specific relationships between key genes, including CKB (D), NTRK3 (E), OTUB1 (F), PCSK1N (G), and VGF (H), and immune cells analyzed based on bulk transcriptomic data of NB patients. (I–M) The associations between five key genes and various immune factors, including receptors (I), major histocompatibility complex (MHC) molecules (J), immune stimulatory factors (K), immunoinhibitor factors (L), and chemokines (M) analyzed by TISIDB database.

The tumor microenvironment is a complex integrated system formed by the interactions of tumor cells with immune cells, extracellular matrix components, various growth factors, inflammatory factors, and unique physical and chemical characteristics. This environment enhances tumor cell proliferation, migration, and immune evasion, thereby promoting tumorigenesis and progression [30]. In NB, the immune microenvironment is notably characterized by limited T cell infiltration, which significantly impacts disease progression and sensitivity to treatments [31]. In this study, we analyzed the relationship between key genes and immune infiltration using CIBERSORT to further explore the molecular mechanisms. The CIBERSORT results for NB patients are presented in Table S6. Our analysis revealed the proportions of immune cell content in NB patients with and without MYCN amplification, as well as the correlations among immune cells (Figure 4A,B). CIBERSORT utilizes a set of immune‐related reference gene expression profiles derived from known immune cell types. During analysis, it compares the gene expression data of the target sample with these reference profiles to estimate the relative proportions of various immune cell types present in the sample [32]. We found significant differences in the levels of memory B cells, resting dendritic cells, activated mast cells, resting mast cells, neutrophils, plasma cells, and resting CD4 memory T cells between the two groups (Figure 4C). These findings suggest that MYCN amplification may promote NB progression by impacting the infiltration of specific immune cell types.

We further investigated the associations between the identified key genes and immune cell populations. The analysis revealed distinct patterns of correlation, suggesting that these genes may play important roles in modulating immune microenvironment in NB. Specifically, CKB exhibited a significant positive correlation with plasma cells and regulatory T cells (Tregs), but negatively correlated with monocytes and resting CD4 memory T cells (Figure 4D). This suggests that CKB may be involved in promoting an immunosuppressive microenvironment through the recruitment or expansion of Tregs, while potentially inhibiting pro‐inflammatory responses mediated by monocytes. NTRK3 showed a significant positive correlation with M1 macrophages (Figure 4E), which are generally considered to have anti‐tumor properties. This finding implies that NTRK3 might contribute to a more immunostimulatory microenvironment, possibly playing a protective role against tumor progression. OTUB1 exhibited significant positive correlations with Tregs and CD8 T cells, while showing negative correlations with M2 macrophages and resting CD4 memory T cells (Figure 4F). The dual presence of Tregs (typically immunosuppressive) and CD8 T cells (typically cytotoxic) suggests a complex regulatory role for OTUB1 in immune balance, potentially influencing the immune escape mechanisms of NB. PCSK1N was positively correlated with activated NK cells and activated CD4 memory T cells, while negatively correlated with resting NK cells and M0 macrophages (Figure 4G). This indicates a potential role in promoting innate and adaptive immune activation, possibly contributing to anti‐tumor immunity. VGF was significantly positively correlated with activated mast cells and activated CD4 memory T cells, and negatively correlated with Tregs (Figure 4H), suggesting that VGF may be linked to a more pro‐inflammatory and less immunosuppressive tumor environment.

Additionally, we obtained correlations between key genes and various immune factors from the TISIDB database (http://cis.hku.hk/TISIDB), including receptors (Figure 4I), major histocompatibility complex (MHC) molecules (Figure 4J), immune stimulatory factors (Figure 4K), immunoinhibitor factors (Figure 4L), and chemokines (Figure 4M). These relationships reinforce the notion that these genes are involved in shaping the immune landscape in NB through both cellular and molecular pathways. Taken together, our findings suggest that these five key genes are intricately linked to the composition and functional state of the immune microenvironment in NB. Their differential associations with immune cell subtypes and immune regulatory factors imply that they may influence tumor progression by modulating immune surveillance, suppression, or activation. These genes may therefore serve not only as prognostic markers but also as potential therapeutic targets for immune‐based interventions in NB.

### 3.5 The Potential Molecular Mechanisms of Key Genes on NB Progression by MYCN Amplification

**FIGURE 5:**
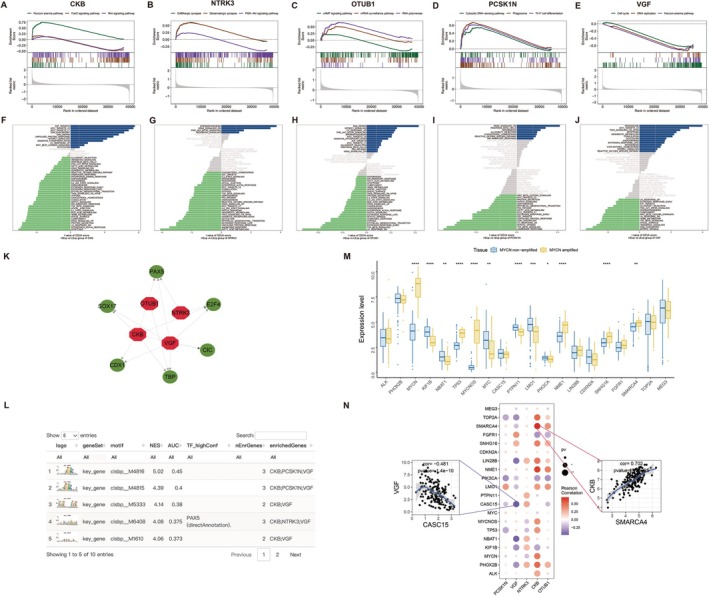
The molecular mechanisms of key genes on NB progression by MYCN amplification. (A‐E) Gene Set Enrichment Analysis (GSEA) of key genes, including CKB (A), NTRK3 (B), OTUB1 (C), PCSK1N (D), and VGF (E) based on NB bulk transcriptome. (F–J) Gene Set Variation Analysis (GSVA) of key genes, including CKB (F), NTRK3 (G), OTUB1 (H), PCSK1N (I), and VGF (J) based on NB bulk transcriptome. (K) Common transcription factors regulating these five key genes and performed enrichment analysis using cumulative recovery curves. (L) Enriched motifs of these key genes and their corresponding transcription factors. (M) Significant intergroup expression differences for genes such as MYCN, KIF1B, NBAT1, TP53, MYCNOS, MYC, PTPN11, LMO1, PIK3CA, NME1, SNHG16, and SMARCA4. (N) Correlation analysis between key genes VGF or CKB and tumor‐related genes.

Next, we investigated the specific signaling pathways involved in the five key genes to explore the molecular mechanisms based on bulk transcriptome analysis. First, GSEA revealed that CKB was enriched in the Fanconi anemia pathway, Wnt signaling pathway, and FoxO signaling pathway (Figure 5A, Figure S2A). NTRK3 was enriched in the GABAergic synapse, PI3K‐Akt signaling pathway, and glutamatergic synapse pathway (Figure 5B, Figure S2B). OTUB1 showed enrichment in the RNA polymerase, mRNA surveillance, and cAMP signaling pathways (Figure 5C, Figure S2C). PCSK1N was enriched in the cytosolic DNA‐sensing pathway, Th17 cell differentiation, and phagosome pathway (Figure 5D, Figure S2D), while VGF was associated with the Fanconi anemia pathway, DNA replication, and cell cycle pathway (Figure 5E and Figure S2E). Additionally, GSVA indicated that high expression of CKB was enriched in pathways such as DNA repair and mTORC1 signaling (Figure 5F). NTRK3 was enriched in pathways like PI3K‐Akt–mTOR signaling and Hedgehog signaling (Figure 5G). OTUB1 was found to enrich pathways such as MYC targets V1 and the P53 pathway (Figure 5H), while PCSK1N was associated with cholesterol homeostasis and reactive oxygen species pathways (Figure 5I). VGF was enriched in pathways including TNFα signaling via NF‐κB and glycolysis (Figure 5J). By integrating the KEGG results from Figure 2 with the pathway analysis of the five key genes, we propose that NTRK3 may influence tumor cell adhesion via PI3K‐Akt signaling. CKB, OTUB1, PCSK1N, and VGF are involved in oxidative phosphorylation and mitochondrial metabolism. CKB, VGF, and OTUB1 regulate cell cycle progression and DNA repair mechanisms. PCSK1N, VGF, and OTUB1 play roles in immune regulation and inflammatory signaling. These connections suggest that the five key genes contribute to NB progression through multiple interrelated pathways, reinforcing the relevance of the KEGG analysis results in Figure 2.

Furthermore, we identified common transcription factors regulating these five key genes and performed enrichment analysis using cumulative recovery curves. The motif with the highest normalized enrichment score (NES: 5.02) was cisbp__M4816. All enriched motifs of the key genes and their corresponding transcription factors, including Paired box 5 (PAX5), E2F transcription factor 4 (E2F4), and SRY‐Box Transcription Factor 17 (SOX17) (Figure 5K,L). The identification of common TFs suggests that these five key genes may be co‐regulated under shared upstream regulatory programs, meaning they likely participate in related biological pathways or functions in NB. PAX5 typically involved in B‐cell lineage commitment, but its aberrant expression in NB may point to ectopic activation of lineage‐inappropriate programs, possibly influencing immune‐related gene expression or tumor plasticity. A well‐known regulator of the cell cycle and proliferation, the enrichment of E2F4 suggests that the key genes may be connected to cell cycle control, consistent with aggressive tumor behavior or high MYCN activity. SOX17 plays a role in developmental signaling and Wnt pathway regulation. Its presence may reflect differentiation state shifts in NB tumors, or even mechanisms of immune evasion or tumor plasticity.

We also accessed the GeneCards database (https://www.genecards.org/) to identify genes associated with NB, extracting the top 20 genes for analysis. This revealed significant intergroup expression differences for genes such as MYCN, KIF1B, NBAT1, TP53, MYCNOS, MYC, PTPN11, LMO1, PIK3CA, NME1, SNHG16, and SMARCA4 (Figure 5M). Correlation analysis showed significant relationships between key genes and tumor‐related genes. Notably, VGF was significantly negatively correlated with CASC15 (*r* = −0.481), and CKB was significantly positively correlated with SMARCA4 (r = 0.702) (Figure 5N). The significant negative correlation between VGF and CASC15 suggests a potential antagonistic relationship in NB biology. This relationship supports the hypothesis that VGF might play a protective role in NB, possibly offering a novel biomarker or therapeutic target for modulating tumor behavior. The strong positive correlation between CKB and SMARCA4 suggests a potential cooperative role in supporting the malignant phenotype of NB cells. It highlights a possible intersection between energy metabolism and epigenetic regulation, which could contribute to tumor progression, adaptation, or therapy resistance. Further wet‐lab experiments and functional validation would help clarify whether these genes act synergistically to drive specific NB phenotypes and whether they represent therapeutic vulnerabilities.

### 3.6 Expression Level and Global Function Analysis of Key Genes in scRNA‐Seq Dataset and Patient Samples

**FIGURE 6:**
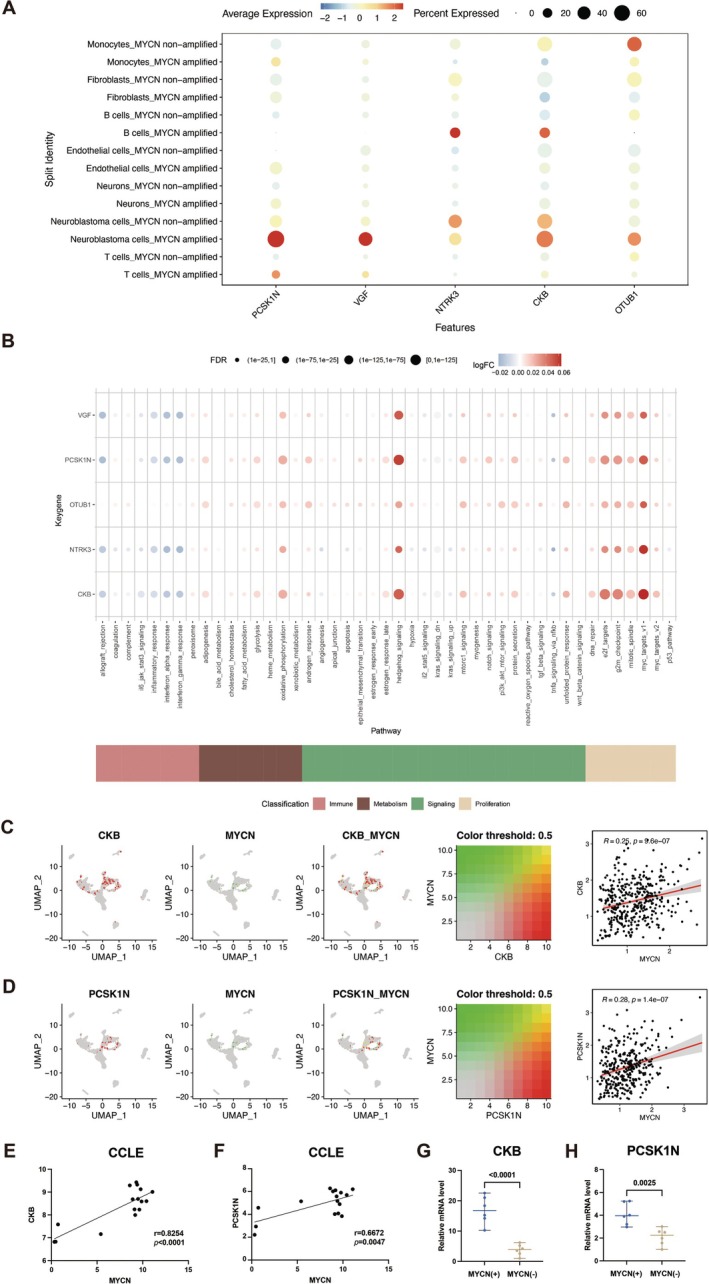
Expression level and global function analysis of key genes in scRNA‐Seq dataset and patient samples. (A) The analyses of gene expression profiles based on patient samples with and without MYCN amplification across seven cell types, including T cells, neuroblastoma cells, neurons, endothelial cells, B cells, fibroblasts, and monocytes. (B) The global analyses of immune and metabolic pathways using single‐cell data, employing the AUCell function. Bubble plots were used to visualize differences in pathway activity related to immune‐metabolic and other signaling pathways. (C, D) The co‐expression patterns and correlation analyses between the expression level of key genes CKB (C) or PCSK1N (D) and tumor‐related MYCN gene. (E, F) The correlation analyses between the expression level of key genes CKB (E) or PCSK1N (F) and tumor‐related MYCN gene in the Cancer Cell Line Encyclopedia (CCLE) database. (G, H) The relative mRNA levels of key genes CKB (G) or PCSK1N (H) in the samples of NB patients with MYCN amplification or not.

We analyzed the expression of key genes across seven cell types, including T cells, NB cells, neurons, endothelial cells, B cells, fibroblasts, and monocytes. These analyses were performed on patient samples with and without MYCN amplification (Figure 6A, Figure S3A,B). The results revealed that the expression levels of PCSK1N, VGF, CKB, and OTUB1 were significantly elevated in the MYCN‐amplified group compared to the non‐amplified group. Conversely, the expression of NTRK3 was significantly reduced in the MYCN‐amplified group. These findings align with our earlier survival analysis, reinforcing the association between these genes and disease prognosis. To further explore the functional implications, we quantified immune and metabolic pathway activity using the AUCell function on single‐cell data. Bubble plots were used to visualize differences in pathway activity related to immune‐metabolic and other signaling pathways. The results indicated that VGF exhibited heightened activity in pathways such as Hedgehog signaling and MYC targets V1, while PCSK1N showed elevated activity in the Hedgehog signaling, MYC targets V1, and other related pathways. Similarly, OTUB1 was highly active in E2F targets, MYC targets V1, and other pathways, while NTRK3 was notably active in G2M checkpoint, MYC targets V1, and Hedgehog signaling pathways (Figure 6B). This is largely consistent with the results of our earlier findings. These observations suggest that the key genes may drive disease progression through modulation of specific oncogenic and metabolic pathways.

Next, we assessed the co‐expression of tumor‐associated progression genes such as ALK, KIF1B, MYCN, NBAT1, and PHOX2B with the five key genes using data from the GeneCards database (https://www.genecards.org/). Co‐expression was visualized across seven cell markers in the scRNA‐Seq dataset. The results revealed that CKB was positively correlated with MYCN expression (*r* = 0.25) (Figure 6C), while PCSK1N also showed a positive correlation with MYCN expression (*r* = 0.28) (Figure 6D). The modest but significant positive correlations suggest that CKB and PCSK1N expression levels tend to increase in MYCN‐high tumor cells, possibly placing them under MYCN regulatory control or within the same oncogenic program. Additionally, CKB was positively correlated with PHOX2B expression (*r* = 0.2) (Figure S3C), and PCSK1N had a similar positive correlation with PHOX2B (*r* = 0.25) (Figure S3D). PHOX2B is a transcription factor critical for autonomic nervous system development. Its co‐expression with CKB and PCSK1N supports that these genes may also play roles in neuronal lineage identity or neuroendocrine differentiation. Furthermore, OTUB1 displayed a strong positive correlation with KIF1B expression (*r* = 0.37) (Figure S3E). These correlations highlight the close relationship between the key genes and tumor progression markers in NB. We further examined the expression of CKB and PCSK1N in the CCLE database and patient samples from our hospital. In 16 NB cell line samples, CKB exhibited a strong positive correlation with MYCN expression (*r* = 0.83) (Figure 6E), and PCSK1N also showed a significant positive correlation with MYCN (r = 0.67) (Figure 6F). Moreover, in clinical samples—comprising 6 MYCN‐amplified cases and 6 non‐amplified cases—both CKB and PCSK1N were significantly overexpressed in patients with MYCN amplification (Figure 6G,H). These findings suggest that CKB and PCSK1N may mark specific subpopulations of tumor cells that are more proliferative, metabolically active, or aggressive, aligning with MYCN and PHOX2B expression profiles.

## 4 Discussion

In this study, we annotated seven cell types in scRNA‐Seq dataset by examining differences between NB patients with MYCN amplification and those without. We observed significant disparities in the marker genes of NB cells between the two groups. Notably, the presence of MYCN amplification alone does not account for all high‐risk cases, complicating the development of universal treatment strategies due to the existence of additional genetic and epigenetic factors [33, 34]. Pathway enrichment analysis of key differentially expressed genes revealed that MYCN primarily influences several oncogenic signaling pathways. These include pathways related to cell adhesion, oxidative phosphorylation, and the cell cycle, as well as pathways associated with sympathetic growth, such as developmental cell growth and the synaptic vesicle cycle [35]. Subsequently, we employed the RSF method to identify crucial genes that significantly impact the aggressive prognosis associated with MYCN. RSF effectively handles censored data and does not assume a specific distribution for survival times [36]. This method captures complex interactions and non‐linear relationships between predictors and survival outcomes, providing more nuanced insights compared to traditional approaches [37]. Additionally, RSF offers measures of variable importance, helping researchers identify which features, such as genetic factors, significantly influence survival outcomes [38]. However, RSF requires a sufficiently large dataset to build reliable models, which can be a limitation in rare diseases or small cohorts. To validate our findings, we conducted survival analyses based on differential expression groups and performed single‐factor, multifactorial, and nomogram prediction model analyses [39]. These results identified five key genes, CKB, PCSK1N, NTRK3, OTUB1, and VGF, are closely associated with prognosis of NB patients, further demonstrating the reliability of RSF approach.

Immunoinfiltration analysis revealed differences in composition and levels of immune cells within the tumor microenvironment between MYCN‐amplified and non‐amplified cases. The identification of memory B cells, resting dendritic cells, activated and resting mast cells, plasma cells, and resting CD4 memory T cells as being associated with MYCN underscores the complex interplay between the immune system and NB biology. The presence of memory B cells and plasma cells indicates an ongoing humoral immune response, suggesting that the immune system is actively recognizing or responding to tumor antigens [40]. This implies a potential for immunotherapeutic approaches, such as vaccines, to enhance anti‐tumor immunity [41]. The role of resting dendritic cells is particularly noteworthy, as they may be crucial in presenting antigens to activate T cell responses against MYCN‐driven tumors [42]. Mast cells, both activated and resting, may play a dual role in tumor progression. While they can contribute to the immune response, they may also promote tumor growth and metastasis through the release of growth factors and cytokines [43]. Additionally, the involvement of resting CD4 memory T cells suggests a potential dysregulation of adaptive immunity in MYCN‐amplified NB, indicating a mechanism by which the tumor evades immune surveillance [44]. Consistently, the negative correlations of CKB with monocytes and resting CD4 memory T cells imply a potential mechanism for immune evasion. NTRK3 may contribute to improved NB prognosis through the antitumor effects of M1 macrophages. The regulation of OTUB1 in Tregs and CD8 T cells highlights its role in promoting an adaptive immune response, while its negative association with M2 macrophages suggests a shift away from a tumor‐promoting environment. Furthermore, the positive relationship of PCSK1N with activated NK and CD4 memory *T* cells reinforces its potential role in fostering anti‐tumor immunity. However, its negative correlation with resting NK cells and M0 macrophages indicates a possible mechanism of immune modulation that favors active immune surveillance [45]. Besides, key genes related to immune factors imply that these genes may be involved in several immunological processes: recruiting or excluding immune cells from the tumor site (e.g., through chemokine signaling); activating or suppressing immune responses, such as by influencing immune checkpoint pathways; altering antigen presentation via MHC molecules, which affects the visibility of tumor cells to the immune system; shaping immune cell phenotypes, for example, by skewing macrophages toward either the pro‐tumor M2 or anti‐tumor M1 subtype [46]. Therefore, the close association of genes with immune factors is particularly relevant in MYCN‐amplified cases, which are often characterized by a highly immunosuppressive microenvironment.

Then GSEA and GSVA results offer insights into functional roles of these identified genes in NB. The enrichment of CKB and VGF in Wnt signaling, FoxO signaling, and DNA replication pathways highlights their critical roles in DNA repair mechanisms and cellular stress responses, where genomic instability drives tumor progression [47]. The link of CKB to mTORC1 signaling also suggests its involvement in metabolic regulation and growth signaling, essential for tumor survival [48]. NTRK3 is enriched in GABAergic and glutamatergic synapse pathways, indicating a connection between neurodevelopmental processes and NB [49]. OTUB1 is enriched in pathways related to RNA polymerase, mRNA surveillance, and cAMP signaling, suggesting its role in regulating gene expression and cellular stress responses. PCSK1N is linked to the cytosolic DNA‐sensing pathway and Th17 cell differentiation, suggesting a dual role in innate immune responses and tumor microenvironment modulation. Its connections to cholesterol homeostasis and reactive oxygen species pathways highlight its involvement in metabolic regulation and oxidative stress management [50]. The identification of enriched motifs for key genes alongside transcription factors like PAX5, E2F4, and SOX17 emphasizes the complex regulatory landscape of gene expression in NB. PAX5, known for its role in B‐cell development, while E2F4 is a key regulator of the cell cycle [51, 52]. The presence of motifs for these transcription factors suggests they may be essential in orchestrating transcriptional programs that support NB cell proliferation and differentiation. The negative correlation between VGF and CASC15 suggests a potential antagonistic relationship worth exploring, as CASC15 has been implicated in cancer progression and may affect tumor growth and metastasis [53]. Conversely, the strong positive correlation between CKB and SMARCA4 points to a potential regulatory relationship, especially since SMARCA4 is involved in chromatin remodeling and transcriptional regulation [54].

We found that there is no direct research on CKB, OTUB1, or PCSK1N in NB. However, a study on Parkinson disease that utilized the NB cell line SY5Y suggested that CKB may be involved in energy metabolism of nerve cells [55]. This aligns with our GSVA analysis that CKB is enriched in oxidative phosphorylation and glycolysis pathways. In contrast, there is well‐documented research on NTRK3 in NB. Marie et al. reported that high expression of NTRK3 inhibits NB progression by inducing apoptosis [56], providing mechanistic insights into our finding that NTRK3 is associated with a favorable prognosis. Additionally, clinical studies have reported that some children with NB develop NTRK3 mutations [57]. Targeted therapies, such as entrectinib, are currently being investigated in clinical trials for both pediatric and adult tumors harboring these mutations [58]. Similarly, the role of VGF in previous studies is consistent with our findings. Research by Lukas et al. identified a potential link between VGF and MYCN amplification [59]. Moreover, a recent study reports that VGF is upregulated in NB [60]. The strong positive correlation between CKB, PCSK1N, and MYCN in NB cell lines, along with the overexpression of both CKB and PCSK1N in MYCN‐amplified samples. CKB may contribute to metabolic process, or cell proliferation that are enhanced in MYCN‐amplified tumors [61]. Involvement of PCSK1N in metabolic and immune‐related pathways may indicate its role in modulating tumor microenvironment, potentially aiding tumor progression and immune evasion in MYCN‐amplified [62].

The dual overexpression of CKB and PCSK1N in MYCN‐amplified cases highlights their potential as biomarkers for this subtype of NB. However, several limitations must be acknowledged. First, our findings are primarily based on bioinformatics analyses. The lack of functional experiments limits our ability to establish a relationship between CKB or PCSK1N and NB. Future studies should incorporate in vitro and in vivo models to explore regulatory mechanisms governing these genes and their impact on MYCN. Another limitation is that our study did not include an independent external validation cohort. While we employed cross‐validation and Monte Carlo simulations to ensure robustness, external validation is still necessary to confirm the generalizability of our findings. Future research should focus on evaluating the therapeutic potential of CKB and PCSK1N inhibition in MYCN‐amplified NB. Small‐molecule inhibitors or RNA‐based therapeutic approaches could be explored to assess tumor response.

## Author Contributions

J.Z. designed the project and wrote the manuscript. X.Z. provided the codes for analyzing single‐cell transcriptome. S.L. and Y.Z. performed the figures creation. J.Z. and D.Z. performed the data analysis in the bulk transcriptome. S.L. reviewed the manuscript. A.L. supervised and funded the project. All authors read and approved the final manuscript.

## Ethics Statement

The studies involving human participants were reviewed and approved by the Medical Ethics Committee of Tongji Hospital, Tongji Medical College, and Huazhong University of Science and Technology (Wuhan, China) (Approval No. 2020‐S207). The parents of the patient provided their written informed consent to participate in this study.

## Consent

All authors have reviewed and approved the final version of this manuscript for publication.

## Conflicts of Interest

The authors declare no conflicts of interest.

## Supporting information


**Figure S1.** Cells filtration, data normalization, and harmony analysis. (A, B) The final violin plot (A) and scatter plot (B) are presented with 3337 cells after data filtration. (C) Data normalization followed by identification of the top 10 genes with highest standardized variance. (D–F) Principal component analysis (PCA) EblowPlot (D), PCA (E), and harmony analysis (F) were performed.

**Figure S2.** The molecular mechanisms of key genes on NB progression by MYCN amplification. (A–E) Gene set enrichment analysis (GSEA) of key genes, including CKB (A), NTRK3 (B), OTUB1 (C), PCSK1N (D), and VGF (E) based on NB bulk transcriptome.

**Figure S3.** Visualization of critical gene expression and global analysis of potential effects. (A, B) The analyses of gene expression profiles across seven cell types, including T cells, neuroblastoma cells, neurons, endothelial cells, B cells, fibroblasts, and monocytes. (C, D) The co‐expression patterns and correlation analyses between tumor‐related genes PHOX2B and key genes CKB (C) or PCSK1N (D). (E) The co‐expression patterns and correlation analyses between tumor‐related genes KIF1B and key genes OTUB1.

**Table S1.** Primer sequences used for RT‐qPCR.

**Table S2.** Raw dataset prior to applying FindAllMarkers.

**Table S3.** Marker genes of NB cells comparing MYCN‐amplified and MYCN non‐amplified groups.

**Table S4.** GO analysis results of marker genes between MYCN‐amplified and MYCN non‐amplified groups.

**Table S5.** KEGG analysis results of marker genes between MYCN‐amplified and MYCN non‐amplified groups.

**Table S6.** CIBERSORT results for immune infiltration analysis.

## Data Availability

The dataset are available in GEO database (https://www.ncbi.nlm.nih.gov/geo/) under the accession number GSE218450, and in the Cancer Genome Atlas Program (TCGA) database (https://www.cancer.gov/ccg/) under the TARGET Neuroblastoma datasets.
