# Supplementary figures and images for: Identification of Crucial Genes Associated With MYCN‐Driven Neuroblastoma Based on Single‐Cell Analysis and Machine Learning
Source: Cancer Med. 2025 Jul 2;14(13):e71008. doi: 10.1002/cam4.71008 (PMC12216506; doi:10.1002/cam4.71008)

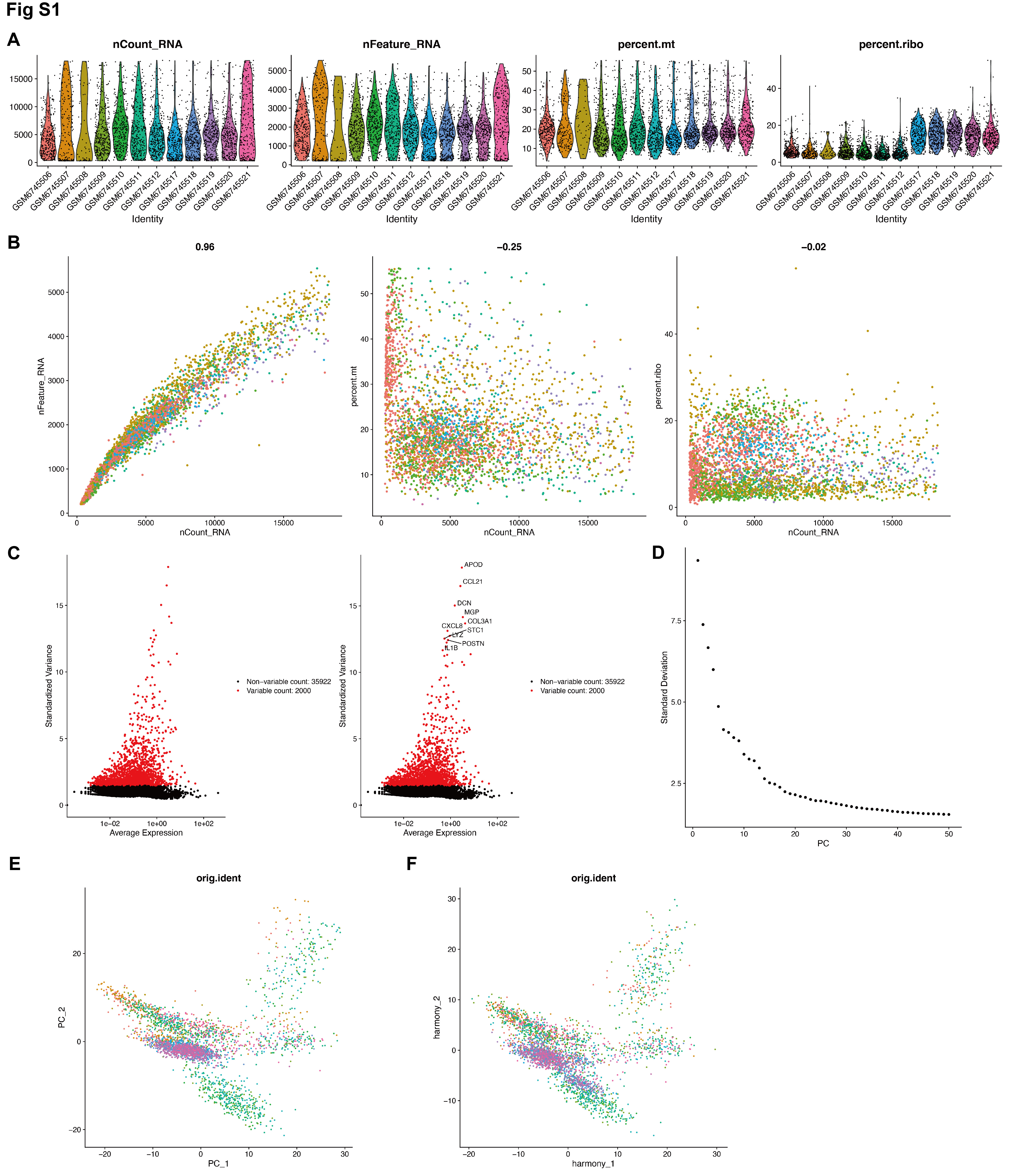

Supplement: Supplementary file 1 — Figure S1. Cells filtration, data normalization, and harmony analysis. (A, B) The final violin plot (A) and scatter plot (B) are presented with 3337 cells after data filtration. (C) Data normalization followed by identification of the top 10 genes with highest standardized variance. (D–F) Principal component analysis (PCA) EblowPlot (D), PCA (E), and harmony analysis (F) were performed. [file CAM4-14-e71008-s005.jpg]

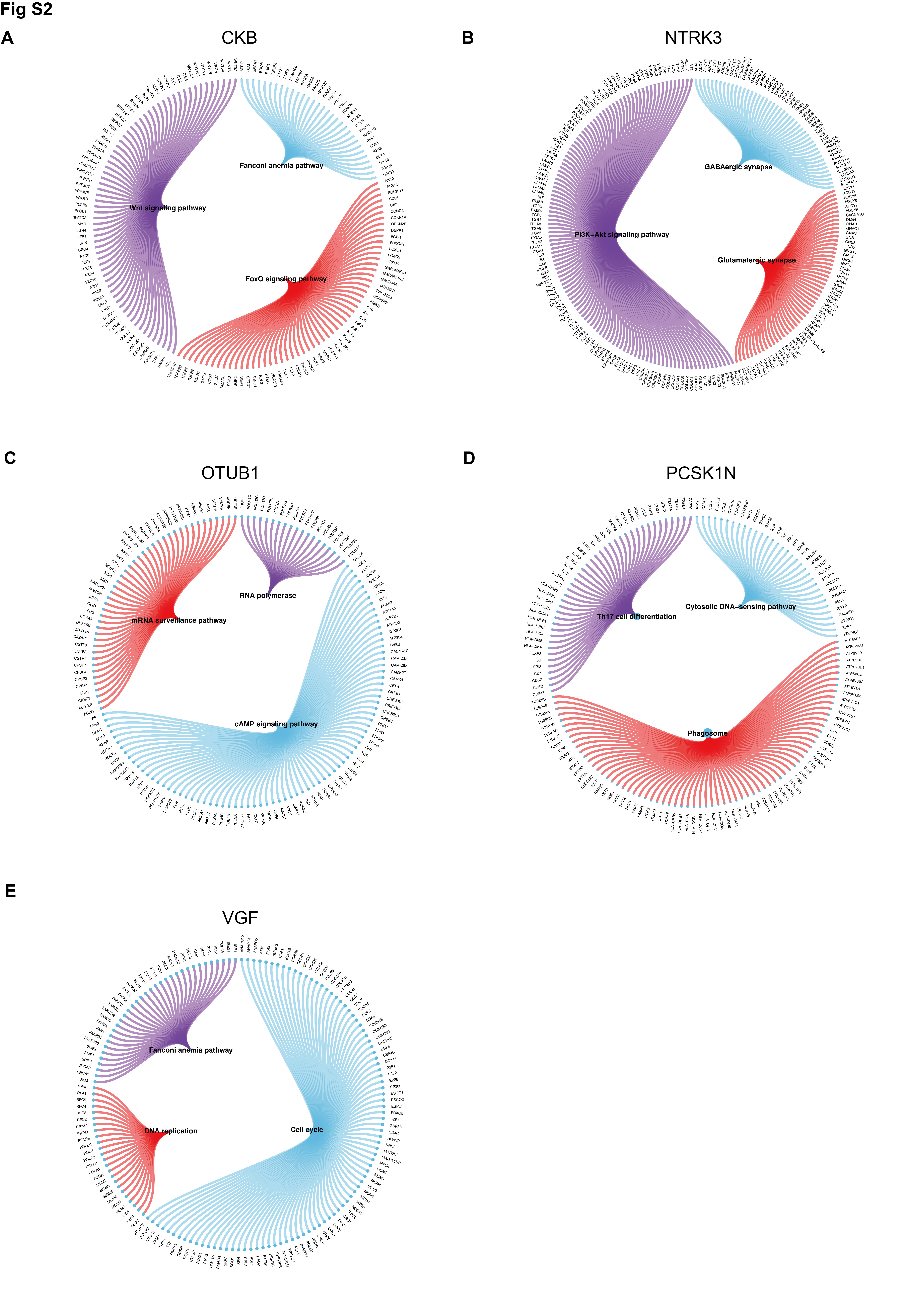

Supplement: Supplementary file 2 — Figure S2. The molecular mechanisms of key genes on NB progression by MYCN amplification. (A–E) Gene set enrichment analysis (GSEA) of key genes, including CKB (A), NTRK3 (B), OTUB1 (C), PCSK1N (D), and VGF (E) based on NB bulk transcriptome. [file CAM4-14-e71008-s006.jpg]

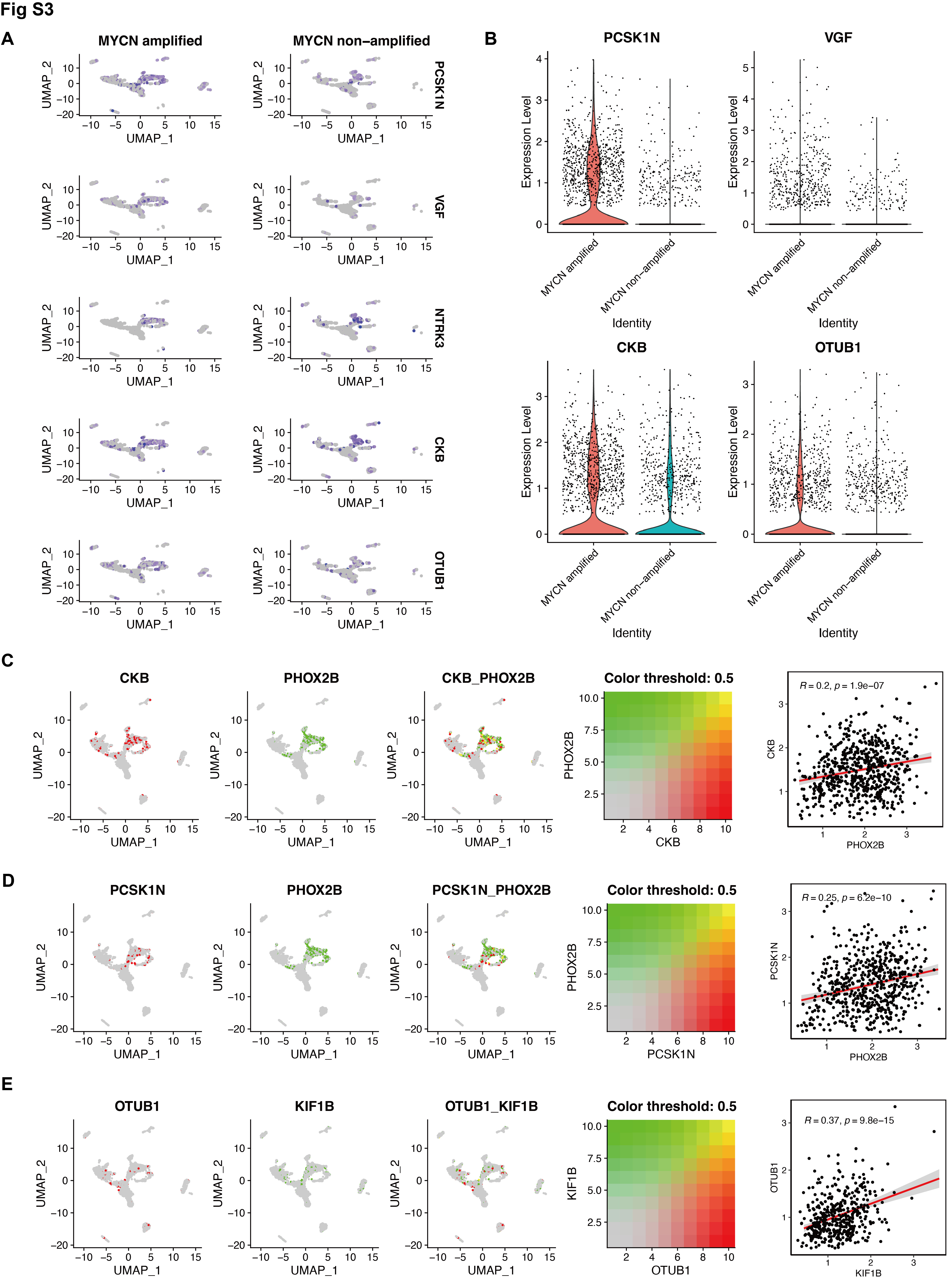

Supplement: Supplementary file 3 — Figure S3. Visualization of critical gene expression and global analysis of potential effects. (A, B) The analyses of gene expression profiles across seven cell types, including T cells, neuroblastoma cells, neurons, endothelial cells, B cells, fibroblasts, and monocytes. (C, D) The co‐expression patterns and correlation analyses between tumor‐related genes PHOX2B and key genes CKB (C) or PCSK1N (D). (E) The co‐expression patterns and correlation analyses between tumor‐related genes KIF1B and key genes OTUB1. [file CAM4-14-e71008-s002.jpg]
